# Molecular and Phenotypic Data Support the Recognition of the Wakatobi Flowerpecker (*Dicaeum kuehni*) from the Unique and Understudied Sulawesi Region

**DOI:** 10.1371/journal.pone.0098694

**Published:** 2014-06-04

**Authors:** Seán B. A. Kelly, David J. Kelly, Natalie Cooper, Andi Bahrun, Kangkuso Analuddin, Nicola M. Marples

**Affiliations:** 1 Department of Zoology, Trinity College Dublin, Dublin, Ireland; 2 Trinity Centre for Biodiversity Research, Trinity College Dublin, Dublin, Ireland; 3 Department of Agrotechnology, Universitas Haluoleo, Kendari, Southeast Sulawesi, Indonesia; 4 Department of Biology, Universitas Haluoleo, Kendari, Southeast Sulawesi, Indonesia; University of California, Berkeley, United States of America

## Abstract

Accurate estimates of species richness are essential to macroecological and macroevolutionary research, as well as to the effective management and conservation of biodiversity. The resolution of taxonomic relationships is therefore of vital importance. While molecular methods have revolutionised taxonomy, contemporary species delimitation requires an integrative, multi-disciplinary approach. Despite boasting a remarkably high level of endemism, the avifauna of the Sulawesi region of Indonesia remains poorly studied. Previous studies of avian diversity in Sulawesi have focussed predominantly on phenotypic characteristics, thus potentially overlooking any genetically distinct lineages. Grey-sided Flowerpecker *Dicaeum celebicum* populations from the Wakatobi archipelago were originally described as a separate species from those on nearby mainland Sulawesi. However, for reasons that remain unknown, the Wakatobi populations were reclassified as a subspecies of the mainland form. Combining estimates of genetic divergence with phylogenetic and morphological analyses, we reassessed the status of Wakatobi populations. Our results describe the Wakatobi populations as a separate species to those on mainland Sulawesi; reproductively isolated, genetically and morphologically distinct. We therefore recommend the reclassification of these populations to their original status of *Dicaeum kuehni* and propose the vernacular name ‘Wakatobi Flowerpecker’. In consideration of our findings and the lack of integrative ornithological research within the Sulawesi region, we believe species richness and avian endemism within the region are underestimated.

## Introduction

Species are the fundamental unit of analysis within ecological, evolutionary and biodiversity research, as well as conservation policy, so accurate estimates of species richness are essential. The presence of erroneously classified species, taxonomic inconsistencies and cryptic diversity are therefore of great significance to these estimates. Accurate species richness data are essential for analysing and understanding patterns of biodiversity [1], as well as for the global designation and conservation of ‘biodiversity hotspots’ [2]. Failure to resolve taxonomic relationships and identify unique lineages may result in the ill-informed distribution of conservation resources, the neglect of certain regions and/or species, and potentially the extinction of species before they are even recognised. Advanced taxonomic knowledge is vital to further understand the complex processes that promote lineage diversification, such as the causes of morphological stasis and mechanisms of mate recognition and choice. Furthermore, such studies may uncover previously unknown ecological relationships [3] and provide further insight into the factors governing them [4].

The understanding of taxonomic relationships has improved dramatically in recent decades, primarily due to improvements in genetic sequencing methods and decreases in associated costs [5], [6]. This has allowed the easy delimitation of genetically and phylogenetically distinct lineages. For example, analyses of the mitochondrial ‘barcoding’ gene COI (cytochrome *c* oxidase subunit I) have revealed numerous genetically divergent bird lineages [7]–[9]. However, while genetic analyses provide one of the simplest and most effective methods for resolving taxonomic relationships, they are best combined with traditional phenotypic analyses [10]. Thus, taxonomic reassessments will be most fruitful if based upon an integrative, multi-disciplinary approach, combining genetic and phylogenetic analyses with comparisons of phenotypic traits, such as morphology, ecology, acoustics and other non-visual mating signals [11]–[13].

While a considerable proportion of the world’s avifauna is known to be incorrectly classified, the birds of Asia are acknowledged as particularly deserving of major reassessment [14]. As well as demonstrating the need for future research into the systematics of Asia’s avifauna, Lohman et al. [8] demonstrated the potential for the discovery of currently unrecognised diversity. The avifauna of the geologically complex Sulawesi region of Indonesia remains poorly studied despite boasting a remarkably high level of endemism (16 endemic genera; 28% species endemism) [15] and facing significant threats of biodiversity loss [16]. Several new species have been described from the Sulawesi region in the last 20 years or so. However, the focus of study has been primarily in the north of the region, with new species described from the Talaud [17], [18], Sangihe [19], [20] and Togian islands [21], [22], as well as northern mainland Sulawesi [23]. Unfortunately, the majority of such studies in the region relied solely on comparisons of phenotypic traits. In the absence of the integration of genetic and phylogenetic analyses, it is possible that studies of avian diversity in Sulawesi have overlooked a significant number of genetically distinct lineages.

The Grey-sided Flowerpecker populations from the Wakatobi archipelago, *Dicaeum celebicum kuehni*, were originally described as a separate species (*D. kuehni*) from those on mainland Sulawesi (*D. c. celebicum*) using only phenotypic criteria [24]. For reasons that remain unclear in the literature, the Wakatobi populations were later subsumed as a subspecies of *D. celebicum*. Here, we employ a multi-disciplinary approach, combining genetic, phylogenetic and phenotypic analyses to investigate geographical variation within the Grey-sided Flowerpecker species and determine whether the Wakatobi populations represent an incorrectly classified, and currently unrecognised, species.

## Materials and Methods

### Ethics Statement

The necessary permits and approvals for this study were obtained from Kementerian Negara Riset dan Teknologi (RISTEK), the Indonesian Institute of Science (LIPI) and Direktorat Jenderal Perlindungan Hutan dan Konservasi Alam (PHKA), the Wakatobi National Park and the Wakatobi Regency. We obtained prior permission from all landowners and no protected species were sampled.

### Sampling

**Figure 1:**
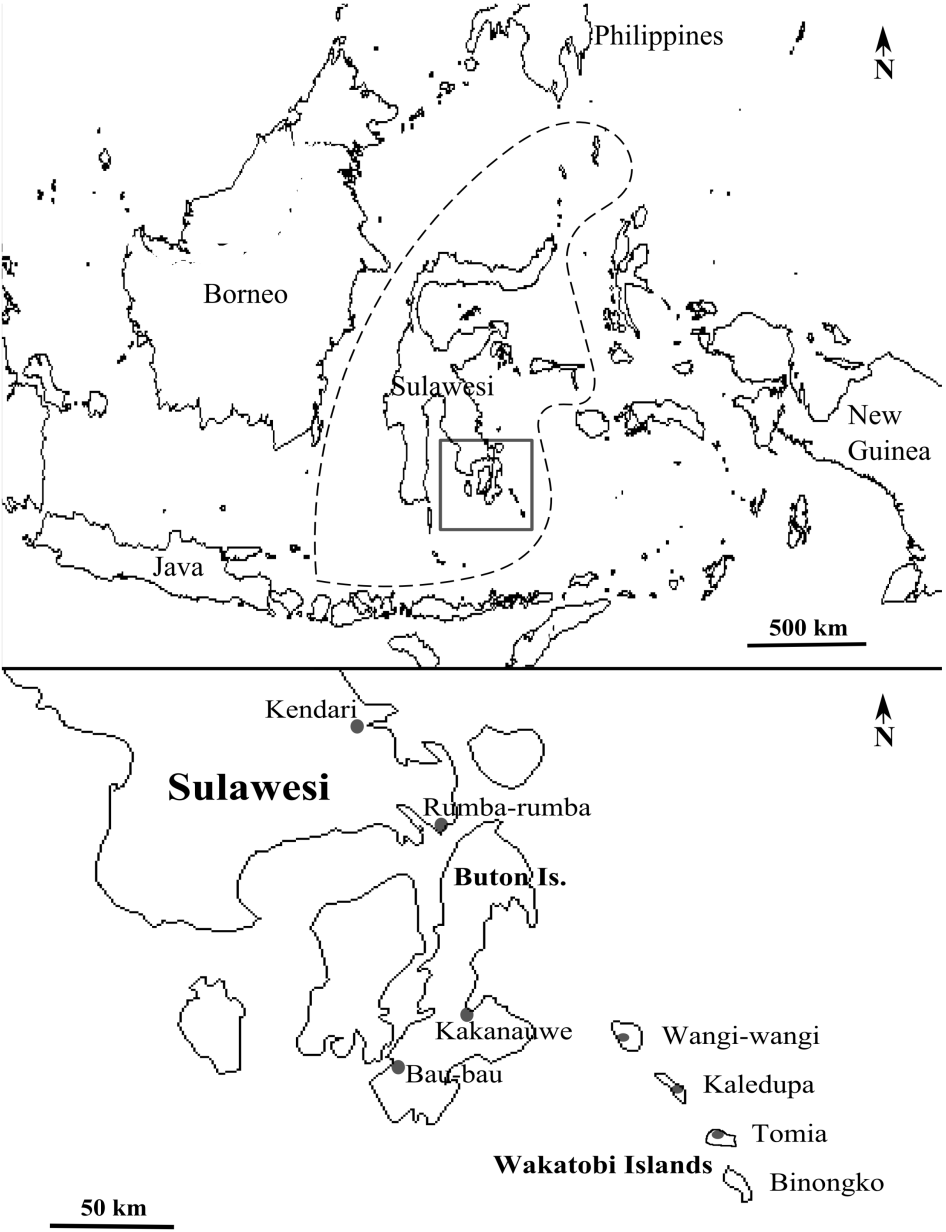
Map showing the Sulawesi region of Indonesia and study sites. Above: location and outline of the Sulawesi region of Indonesia (dashed lines) and the study area (square). Below: enlarged view of the study area showing peninsular south-east Sulawesi, Buton and the Wakatobi Islands; closed circles indicate sampling sites.

For this study, we sampled Grey-sided Flowerpeckers from seven sites across the Wakatobi archipelago, Buton Island and the south-eastern peninsula of Sulawesi (Figure 1). The Wakatobi islands are located off the coast of peninsular south-east Sulawesi, approximately 27 km from Buton island, the nearest landmass representative of mainland Sulawesi [25]; thus, from herein, when Sulawesi is referred to, it includes Buton island. In total, 58 Grey-sided Flowerpeckers (16 female and 36 male) were mist-netted from Buton and South-east Sulawesi (N = 29) and the Wakatobi islands (N = 29) during the months of July, August and September in various years between 1999 and 2012. When possible, each bird was assessed for age (adult or juvenile) and sex. The following morphometric measurements were taken for each individual (by NMM only): wing length (maximum chord), weight, bill length (to skull), bill depth (at deepest), tail length (standard), tarsus length (‘minimum’) and total head length (head plus bill) [26]. Prior to release, a small cluster of contour feathers were plucked from the flank of each bird.

### DNA Sequencing

DNA was extracted from feathers using a Qiagen DNeasy Blood and Tissue Kit (Qiagen, California, USA). Extraction followed the manufacturer’s protocol except for the final step where bound DNA was eluted twice from the spin column using 150 µl of Buffer AE instead of 200 µl. A 672 bp region of the COI gene was amplified using barcoding primers Passer-F1 and Passer-R1 [27]. The complete mitochondrial gene ND3 (NADH dehydrogenase subunit 3; 351 bp) was amplified using primers L10755-F and H11151-R [28] which have proved successful in other flowerpecker studies [29], [30]. All polymerase chain reaction (PCR) amplifications were performed in 20 µl reactions consisting of 11.3 µl double-distilled H_2_0, 0.4 µl 10 mM deoxynucleoside triphosphates (dNTPs), 2 µl 10×PCR reaction buffer (MgCl_2_), 1.2 µl 50 mM MgCl_2_, 1 µl 10 µM forward primer, 1 µl 10 µM reverse primer, 0.1 µl Taq polymerase and 3 µl template DNA. All COI reactions were amplified under the following thermal cycler conditions: 4 min at 94°C followed by 35 cycles of 1 min at 94°C, 1.5 min at 58°C and 1.5 min at 72°C, finishing with 5 min at 72°C (adapted from [7]). Thermal cycling for ND3 ran for 45 cycles instead of 35 to ensure detection of even trace amounts of DNA and the second step of this cycling was lowered from 58°C to 55°C [adapted from [28]]. PCR products were then cleaned using ExoSAP protocol [31], cycle-sequenced with BigDye Terminator v3.1 Cycle Sequencing Kit (Applied Biosystems, California, USA), subsequently cleaned with BigDye XTerminator Purification Kit (Applied Biosystems, California, USA) and sequenced in both directions on an Applied Biosystems 3130xl genetic analyzer. All sequences were submitted to GenBank [32] under accession numbers JX067909–JX067924.

### Phylogenetic and Genetic Analyses

A number of COI and ND3 sequences from *Dicaeum* and *Nectarinia* (outgroup) species were sourced from GenBank [32] to facilitate more robust phylogenetic analyses. The accession numbers of all sequences included can be seen in Table S1. The outgroup for each phylogeny was composed of *Nectarinia* sunbirds (Nectariniidae), close relatives of the flowerpeckers (Dicaeidae) [33]. Numerous sequences were included in the outgroup to generate a robust phylogenetic hypothesis [34]. COI and ND3 sequences were aligned separately using ClustalW multiple alignment in BioEdit v.7.1.3.0 [35] and concatenated using Mesquite v.2.75 [36]. While some partial tRNA fragments flanking the ND3 gene were sequenced, these sequences were not included in our analyses. Using MEGA v.5.0 [37], a Hasegawa-Kishino-Yano model with five gamma categories and the presence of invariant sites (HKY+5Γ+I) was chosen as the most suitable evolutionary model to infer the phylogeny of our combined genes. To avoid the over-parameterisation of our models we excluded invariant sites from our analyses. We carried out Bayesian phylogenetic inference of haplotypes using Mr. Bayes v.3.2.2 [38]. Our model parameters included a HKY substitution model, two independent runs consisting of four chains of five million generations, sampling every 1,000 generations and a burn-in of 25%. Convergence in runs was accepted when the average standard deviation in split frequencies reached 0.01 [39] and the effective sample size of model parameters exceeded 200 [40]. Maximum likelihood analysis was carried out in RaxML v.8 [41], using a GTRCAT substitution model with five distinct gamma categories, partitioned by codon position and run for 1,000 rapid bootstrap replicates.

Using COI sequences, pairwise comparisons were carried out in MEGA v.5.0 [37] to calculate maximum, minimum and mean uncorrected proportional genetic distances (*p*-distances) within and between Wakatobi and Sulawesi populations. These were then compared to known levels of between species genetic divergence [42].

### Morphological Analyses

We used two-way ANOVA models to compare populations from the Wakatobi archipelago to those on Sulawesi for each of the following morphological traits: wing length, weight, tarsus length, tail length, skull length (total head minus bill length), and bill length and depth. All data were log_10_-normalized prior to analysis. As Grey-sided Flowerpeckers are sexually dimorphic [33], we included sex as a fixed effect in all models to control for this. We applied sequential Holm-Bonferroni corrections to correct for multiple comparisons [43]. Our analyses were restricted to adult birds, as juvenile birds were netted only on the Wakatobi archipelago. All analyses were run in R v.3.0.1 [44].

## Results

### Phylogenetic and Genetic Analyses

**Figure 2:**
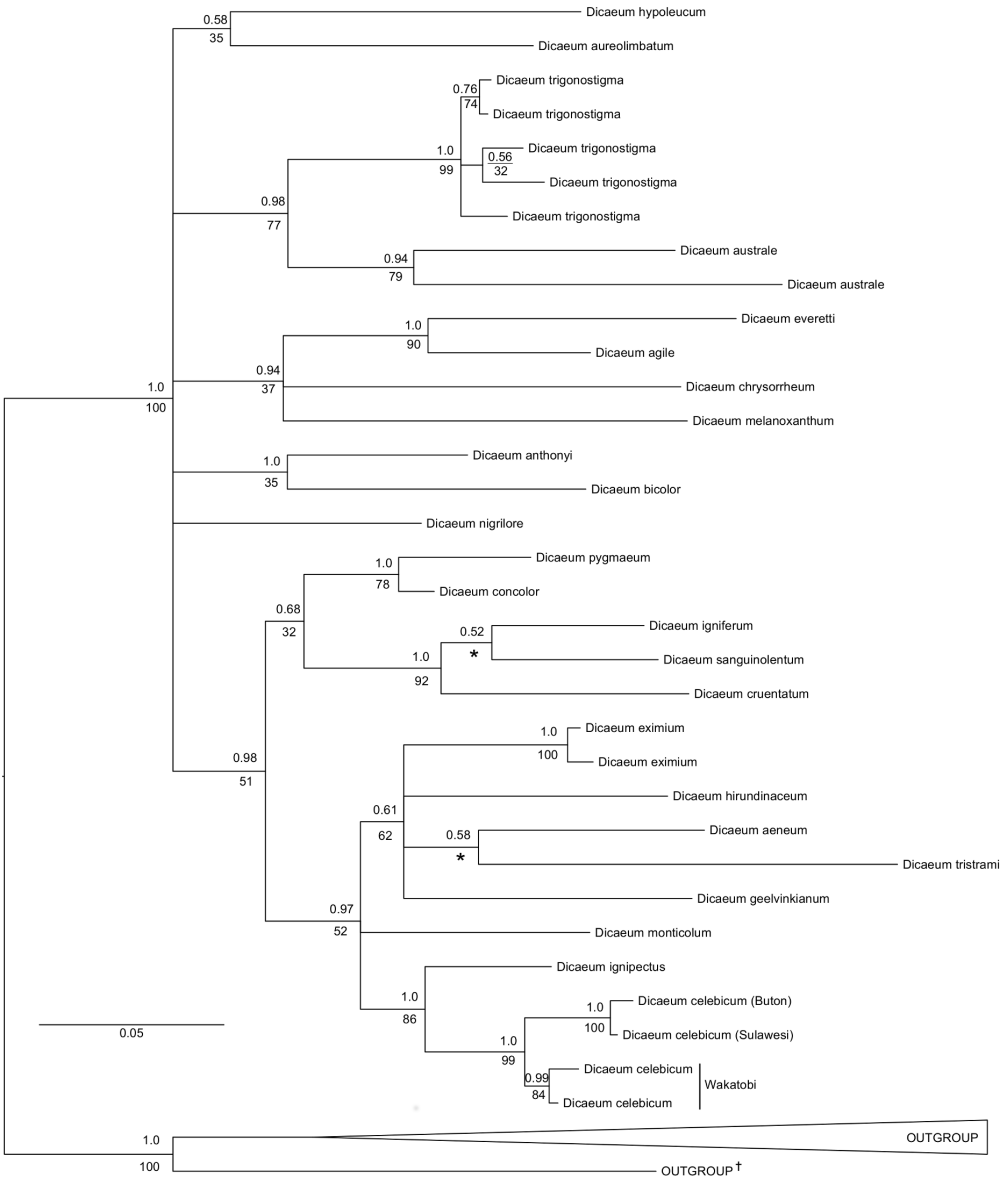
Bayesian consensus tree for concatenated COI and ND3 haplotypes, showing Bayesian posterior probabilities (above) and bootstrap values from our maximum likelihood analysis (below) for each node, with outgroup lineages collapsed. Nodes not present in our maximum likelihood analysis are marked with an asterisk. The accession numbers for all sequences included are given in Table S1. ^†^This outgroup branch represents *Nectarinia sperata* which was monophyletic with respect to the rest of the outgroup and whose position with the genus remains uncertain [53].

Results from our maximum likelihood and Bayesian analyses of concatenated COI and ND3 haplotypes (Figure 2) described the Wakatobi and Sulawesi populations of Grey-sided Flowerpecker as reciprocally monophyletic, comprising two distinct evolutionary lineages. This split between Wakatobi and Sulawesi populations was strongly supported in both our maximum likelihood and Bayesian analyses (see Figure 2). Wakatobi and Sulawesi populations shared none of the same COI or ND3 haplotypes, suggesting the two populations were reproductively isolated. Furthermore, the branch lengths for Sulawesi populations were much longer than those of Wakatobi populations. We refrained from collapsing lineages with poor bootstrap (<50%) or Bayesian support (<0.95 posterior probability) to allow for the visualisation and comparison of divergent lineages within current taxonomic species. However, when we did collapse lineages with poor bootstrap support, Wakatobi and Sulawesi populations remained reciprocally monophyletic. Genetic divergence between the Wakatobi and Sulawesi populations for COI ranged from 2.53–2.83% and mean divergence within populations was 0.26%. Within the *Dicaeum* genus (ingroup), 159/351 (45.3%) of sites were variable and 118/351 (33.6%) sites were parsimony-informative for ND3, while 174/651 (26.8%) and 126/651 (19.4%) of sites were variable parsimony-informative for COI, respectively.

### Morphological Analyses

**Figure 3:**
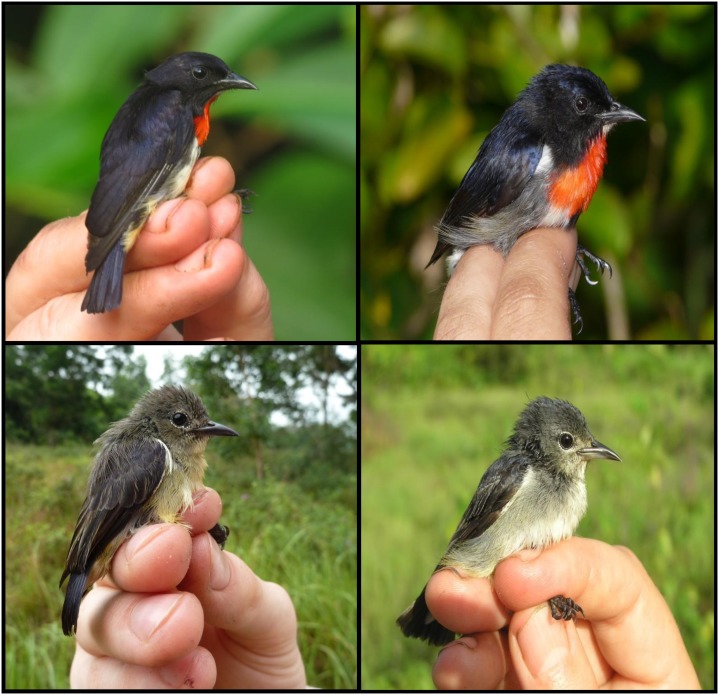
A comparison of overall plumage characteristics between male (top row) and female (bottom row) flowerpeckers from mainland Sulawesi (left column) and the Wakatobi archipelago (right column).

Birds from the Wakatobi islands had significantly longer wings (*F*_1,49_ = 152.76, *p*<0.001), bills that were longer (*F*_1,47_ = 201.67, *p*<0.001) and deeper (*F*_1,39_ = 17.02, *p*<0.001), longer tarsi (*F*_1,44_ = 151.31, *p*<0.001), longer skulls (*F*_1,45_ = 58.95, *p*<0.001), longer tails (*F*_1,39_ = 130.12, *p*<0.001) and were heavier (*F*_1,48_ = 136.93, *p*<0.001) than birds from mainland Sulawesi. These results control for the effects of sex and multiple testing. Morphological data are summarized in Table 1. Additionally, we observed the same differences in plumage between populations as originally described by Hartert [24]. Males from the Wakatobi archipelago have distinct steel-blue upperparts with a purplish gloss, compared to the dark purple upperparts of males from Sulawesi. The red colouration extends farther down the breast of Wakatobi males and they have lighter, greyer flanks and abdomen compared to Sulawesi males (see Figure 3). Consistent with other sources [24], [33], we noted no major plumage differences between females from Wakatobi and Sulawesi populations (see Figure 3).

**Table 1:** Morphological comparisons of adult male and female birds between Sulawesi and Wakatobi populations showing the mean and standard error for each character.

	Sulawesi		Wakatobi	
Character*	Male	Female	Male	Female
Wing length	47.6±1.5 (*N = 20)*	44.3±1.3 (*N* = 9)	52.3±1.1 (*N* = 16)	48.8±1.5 (*N* = 7)
Weight	5.8±0.4 (*N = 20)*	5±0.4 (*N* = 9)	7±0.4 (*N* = 16)	7.2±0.8 (*N* = 7)
Bill length	11±0.4 (*N = 19)*	10.8±0.6 (*N* = 9)	13.1±0.5 (*N* = 15)	12.8±0.4 (*N* = 7)
Bill depth	2.7±0.1 (*N = 17)*	2.6±0.2 (*N* = 9)	2.8±0.2 (*N* = 9)	2.8±0.2 (*N* = 7)
Skull length	11.9±0.4 (*N = 17)*	11.4±0.6 (*N* = 9)	13.2±0.9 (*N* = 15)	12.7±0.3 (*N* = 7)
Tarsus length	11.2±0.5 (*N = 17)*	10.7±0.5 (*N* = 9)	12.8±0.4 (*N* = 14)	12.6±0.4 (*N* = 7)
Tail length	24±1 (*N = 17)*	21.8±1 (*N* = 9)	27.2±0.8 (*N* = 9)	26.1±0.9 (*N* = 7)

*All characters are presented in units of millimetres except weight which is presented in grams.

## Discussion

Our combined results from genetic, phylogenetic and morphological analyses, identify the Wakatobi populations of Grey-sided Flowerpecker as a distinct species, separate from the populations of mainland Sulawesi. Wakatobi and Sulawesi populations share no COI or ND3 haplotypes; this is one of the simplest tests of species identification using DNA barcodes [9], [42]. This also suggests that there is no gene flow between the populations and that they are reproductively isolated from one another, a defining criterion of species under the biological species concept [45]. The level of divergence in COI sequences between Wakatobi and Sulawesi populations (2.53–2.83%) is within the range of divergence between species (2.7%) observed by Hebert et al. [42] in a landmark study of over 260 North American bird species. Considering this, we believe the extent of genetic divergence between the two populations represents a separation at the species level.

Our morphological results support Hartert’s [24] original findings that Wakatobi populations are significantly larger than those on Sulawesi for various traits. Furthermore, this morphological divergence is suggestive of major ecological differences between these populations. The increases in tarsus length and bill size (length and depth) seen in Wakatobi populations are associated with changes in foraging and feeding ecology, respectively, both of which are suggestive of niche expansion [46], [47]. The observed increases in body size (which can be indexed from wing length and weight) may be due to reduced interspecific competition, increased intraspecific competition (with comparatively higher population densities) and/or variation in energetic constraints and physiological optimisation [46]–[49]. These results suggest that despite being separated by only 27 km, there are likely to be major differences in the selective pressures acting upon the populations of small passerines on mainland Sulawesi and the Wakatobi archipelago. Behavioural and ecological studies of both Wakatobi and Sulawesi populations would help provide further insight into these suggested ecological differences.

Phylogenetic analyses of COI and ND3 sequences have provided useful corroborating evidence in the delimitation of other bird species, including other *Dicaeum* flowerpeckers [8], [29], [30]. Some basal bootstrap and Bayesian support values from the inferred COI and ND3 phylogeny (Figure 2) are rather low and the overall structure of the phylogeny is thus not likely to accurately reflect broad relationships within the *Dicaeum* genus. A lack of sampling and gene sequencing of *Dicaeum* species is likely to be a contributing factor in this regard. Interestingly, the difference in branch lengths between Wakatobi and Sulawesi populations suggest Sulawesi lineages have experienced more genetic change since the populations split. Furthermore, the branch lengths separating Wakatobi and Sulawesi lineages are similar to those separating *D. trigonostigma* populations, a lineage which is known to contain currently undescribed species [8].

When taken independently, our genetic, phylogenetic and morphological results provide limited evidence of divergence, but when combined they allow robust conclusions to be drawn regarding species delimitation (e.g. [12], [13], [50]). Therefore, in consideration of our findings, previously described plumage differences [33] and a lack of justification for the departure from Hartert’s original species description [51], we advocate the reclassification of the Wakatobi populations to their original status as *Dicaeum kuehni* [24], a species endemic to the Wakatobi archipelago. We propose the vernacular name ‘Wakatobi Flowerpecker’ for *D. kuehni*. The recognition of *D. kuehni* as an endemic species will require BirdLife International to reassess their current categorisation of the Wakatobi archipelago as an Important Bird Area (ID184) [52]. The Wakatobi currently hosts five endemic subspecies [33], two of which were also originally described as species by Hartert [24]; therefore, in light of our findings, we believe these populations warrant re-examination. Furthermore, the Banggai and Sula, Sangihe and Talaud islands each support an endemic subspecies of *D. celebicum*, all of which deserve re-assessment using the same integrative approach as this study. Our study provides further evidence of unrecognised diversity and ‘over-lumping’ within the Sulawesi and Asian avifauna. Considering this, as well as the paucity of research, lack of multi-disciplinary studies of avian diversity and the complex geological history of the Sulawesi region, there are likely to be many more taxonomic relationships to be resolved and species to be described.

## Supporting Information

Table S1 Accession numbers of sequences used in this study’s phylogenetic analyses.
(XLSX)
